# Broomrape Weeds. Underground Mechanisms of Parasitism and Associated Strategies for their Control: A Review

**DOI:** 10.3389/fpls.2016.00135

**Published:** 2016-02-19

**Authors:** Mónica Fernández-Aparicio, Xavier Reboud, Stephanie Gibot-Leclerc

**Affiliations:** ^1^INRA, UMR 1347 AgroécologieDijon, France; ^2^Agrosup-Dijon, UMR 1347 AgroécologieDijon, France

**Keywords:** integrated pest management, *Orobanche*, *Phelipanche*, parasitism, germination, haustorium, plant recognition, seed bank

## Abstract

Broomrapes are plant-parasitic weeds which constitute one of the most difficult-to-control of all biotic constraints that affect crops in Mediterranean, central and eastern Europe, and Asia. Due to their physical and metabolic overlap with the crop, their underground parasitism, their achlorophyllous nature, and hardly destructible seed bank, broomrape weeds are usually not controlled by management strategies designed for non-parasitic weeds. Instead, broomrapes are in current state of intensification and spread due to lack of broomrape-specific control programs, unconscious introduction to new areas and may be decline of herbicide use and global warming to a lesser degree. We reviewed relevant facts about the biology and physiology of broomrape weeds and the major feasible control strategies. The points of vulnerability of some underground events, key for their parasitism such as crop-induced germination or haustorial development are reviewed as inhibition targets of the broomrape-crop association. Among the reviewed strategies are those aimed (1) to reduce broomrape seed bank viability, such as fumigation, herbigation, solarization and use of broomrape-specific pathogens; (2) diversion strategies to reduce the broomrape ability to timely detect the host such as those based on promotion of suicidal germination, on introduction of allelochemical interference, or on down-regulating host exudation of germination-inducing factors; (3) strategies to inhibit the capacity of the broomrape seedling to penetrate the crop and connect with the vascular system, such as biotic or abiotic inhibition of broomrape radicle growth and crop resistance to broomrape penetration either natural, genetically engineered or elicited by biotic- or abiotic-resistance-inducing agents; and (4) strategies acting once broomrape seedling has bridged its vascular system with that of the host, aimed to impede or to endure the parasitic sink such as those based on the delivery of herbicides via haustoria, use of resistant or tolerant varieties and implementation of cultural practices improving crop competitiveness.

## Introduction

The broomrapes are obligate plant-parasitic plants from the genera *Orobanche* and *Phelipanche* in the Orobanchaceae family (Bennett and Mathews, 2006; Tank et al., 2006; Joel, 2009). Due to their achlorophyllous nature, broomrapes are constrained to obtain their nutritional resources by feeding off other plants using the haustorium, an organ unique in parasitic plants through which the parasite diverts water and nutrients from the host (De Candolle, 1813; Kuijt, 1969; Musselman and Dickison, 1975; Westwood, 2013). The majority of broomrape species are botanical wonders parasitizing wild host plants in natural ecosystems. However, seven broomrape species, *Orobanche crenata*, *O. cernua*, *O. cumana*, *O. foetida*, *O. minor*, *Phelipanche aegyptiaca*, and *P. ramosa* have specialized on attacking crops causing trouble in agriculture along Mediterranean, central and eastern Europe, and Asia (Parker, 2009). The crops affected depend on the host range of the broomrape species considered but in general, those in the Asteraceae, Brassicaceae, Apiaceae, Fabaceae, or Solanaceae such as sunflower, oilseed rape, carrot, faba bean, or tomato among many others, sustain the major attacks (Parker and Riches, 1993). The damage induced in the crop by broomrape parasitism differs for each broomrape-host association. In general, parasitized crops suffer from reductions in total biomass at the greatest expense to the reproductive tissue (Barker et al., 1996; Manschadi et al., 1996; Lins et al., 2007). In some crops, the biomass loss equals to that accumulated by the parasite indicating that damage in the crop is directly attributed to the parasitic sink activity (Barker et al., 1996; Manschadi et al., 1996; Hibberd et al., 1998). However, in other broomrape-crop associations the damage induced by broomrape extends beyond assimilate diversion. In those cases, broomrape displays a pathogenic nature promoting disease in the crop mainly through negative effects on the crop photosynthetic machinery and hormonal balance (Stewart and Press, 1990; Mauromicale et al., 2008).

There are not figures based on rigorous data for the total area affected by broomrape weeds (Parker, 2009). Sauerborn (1991) estimated that 16 million ha in Mediterranean and West Asia regions risked being infested. In this regard, France is doing valuable work through the Technical Center for Oilseed Crops and Industrial Hemp, Terresinovia, where a nationwide survey of infested fields is actualized online on real time by the farmers with new cases emerging every year and recently toward new regions such as the French Centre region^1^ Several studies suggest that large areas of new territory are at risk of invasion by broomrape (Mohamed et al., 2006; Grenz and Sauerborn, 2007), and in fact, invasions in completely new regions are already emerging in countries such as Spain, UK, France, Algeria, Ethiopia, Egypt, Sudan (Reda, 2006; Babiker et al., 2007; Babiker, 2008; Rubiales et al., 2008; Abu-Irmaileh and Labrada, 2009; Parker, 2014).

Several factors contribute to the fact that broomrape weeds remain an uncontrolled agricultural problem. Control strategies designed for non-parasitic weeds such as cultural and chemical methods do not necessarily achieve the required level of control for broomrape due to its mixed traits as weed and as root parasite. Biological traits in broomrape such as achlorophyllous nature, underground parasitism, the physical connection and growth synchronization with the crop, and the exclusive uptake of resources via crop vascular system rather than from the soil make broomrape control a challenging agricultural task. In addition, the biological similarity between host and parasite characterizing broomrape-crop interactions is higher than in other plant pathosystems, which complicates the development of selective methods to control broomrape, without harmful effect in the crop from which it is feeding (Eizenberg et al., 2006; Hearne, 2009; Yoder and Scholes, 2010; Pérez-Vich et al., 2013).

**FIGURE 1:**
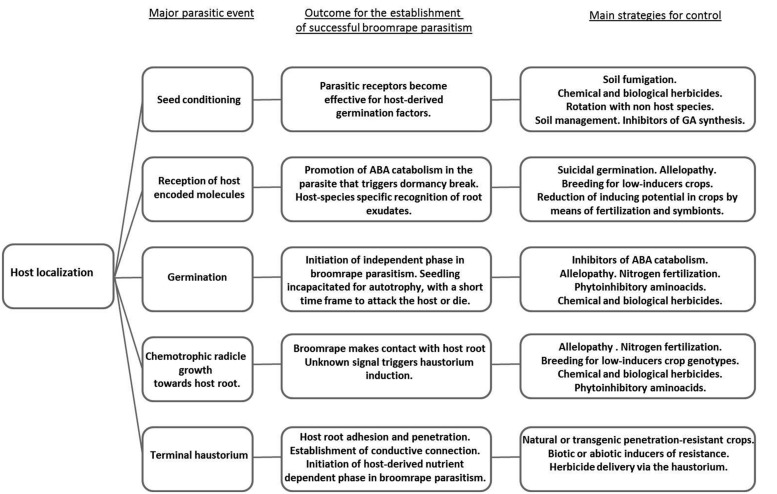
**Flowchart showing major underground parasitic events developed by broomrape weeds on susceptible crops and the control strategies that successfully target them**.

Besides the difficulty of selectively controlling broomrape in the form of host-attached parasite, eradication of broomrape seed bank is extremely difficult due to prolific production of parasitic seeds, their easy dispersal, physiological dormancy, seed longevity, and germination synchronized with specialized range of host cultivation. New infestations can occur through the use of contaminated seeds or machinery and their prevention is essential. Despite of this fact, Seed Certification Services in some of the countries affected, do not include in their certification standards, inspection of crop seed samples for broomrape inoculum. Effective broomrape control should target the underground mechanisms of crop parasitism in order to meet both the short-term productivity expectations of the farmer and reduction of soil bank in the long run (**Figure 1**). Promising new control strategies have been investigated though the majority of them are under development or remain as prototypes to which farmers have not access. Although some examples of successful control do exist for some crops, the majority of commercially available control methods are either not fully effective or not applicable to many of the affected crops, especially in the case of low-input crops (Joel, 2000).

This paper reviews relevant facts about the biology of broomrape weeds, the key mechanisms they employ to attack crops and the control methods already developed or in development that directly target those mechanisms.

## Specialized Mechanisms in Broomrape Weeds for a Parasitic Mode of Life

The evolution from autotrophic to heterotrophic mode of nutrition carried a reduction of the main broomrape vegetative organs toward vestigial versions, non-functional for autotrophy. Root system in mature broomrape plants is reduced to short adventitious parasitic roots with functions of anchorage and stabilization in the soil and their leaves are reduced to small achlorophyllous scales (Parker and Riches, 1993). In return they develop haustoria to feed off other plants (Kuijt, 1969; Musselman and Dickison, 1975). The haustorium is the key feature of plant parasitism which has evolved independently at least 11 times in angiosperms (Barkman et al., 2007; Westwood et al., 2012; Yang et al., 2015). Haustorium allows broomrape to attack crops by successive functions, first as host-adhesion organ, and subsequently as invasive organ toward host vascular system where finally establishes vascular continuity allowing the parasite to withdraw water and nutrients from the host (Riopel and Timko, 1995; Joel, 2013). The terminal haustorium develops at the apex of the seedling radicle upon host recognition (Musselman, 1980; Joel and Losner-Goshen, 1994). This is a short and delicate stage where the parasite either connects with the host or dies due to nutrient exhaustion. As a consequence of the high risk of establishment failure in the seedling, broomrapes have evolved germination strategies that “predict” establishment potential based on host chemodetection (Vaucher, 1823). In the following sections we describe the key developmental stages in the subterranean broomrape life cycle. These stages constitute sites of broomrape metabolism at which it is possible to design successful strategies to inhibit its sophisticated parasitism.

### Host Localization

#### Seed Conditioning

Broomrape seed bank remains viable in the soil for many years until germination is triggered by the coincidence of several physical and chemical factors that are indicative of environmental conditions for successful seedling establishment: i.e., the nearby growth of a host plant in a physiological stage susceptible for broomrape invasion and subsequent parasitic reproductive growth (Linke and Saxena, 1991; López-Granados and García-Torres, 1996, 1999). Although hard seed coat has been described as dormancy mechanism in newly formed broomrape seeds (López-Granados and García-Torres, 1996), water uptake and imbibition are performed quickly by mature seeds through the micropyle without the need of scarification (Bar-Nun and Mayer, 1993; Joel et al., 2012). Broomrape seed bank presents annual cycles of non-deep physiological dormancy induced by seasonal changes in climatic conditions. In non-parasitic plants, physiological dormancy can be relieved through stratification but in the case of broomrape weeds, two consecutive processes are required to release dormancy: an environment-dependent first step of warm stratification called the conditioning phase, and a host-dependent second step of chemodetection. The first step of conditioning promotes in the parasitic seed receptors the required sensitivity for the second step of host detection (Musselman, 1980; Kebreab and Murdoch, 1999; Lechat et al., 2012, 2015; Murdoch and Kebreab, 2013).

The length and temperature required to promote seed conditioning depends on the broomrape species but are usually described under laboratory conditions in a range of 4–12 days at a temperature of 19–23°C, in dark and humid conditions (Kebreab and Murdoch, 1999; Gibot-Leclerc et al., 2004; Lechat et al., 2012). This treatment in the lab mimics the soil conditions in climatically suitable regions for broomrape such as Mediterranean non-irrigated agrosystems where the onset of warm and wet season coincides with the growth of juvenile stages of many annual crops (López-Granados and García-Torres, 1996; Grenz and Sauerborn, 2007). It is important for broomrape to initiate parasitism in young crops otherwise host reproductive organs in the rapid seed-filling stage will be able to endure a delayed parasitism by establishing a stronger competition with parasitic sinks (Manschadi et al., 1996; Fernández-Aparicio et al., 2009a, 2012a). Therefore broomrape seeds timely gain sensitivity for host chemodetection by means of conditioning (López-Granados and García-Torres, 1996). In absence of host detection the continuation of wet conditions allows broomrape seeds to enter again in deeper levels of dormancy, from which they will emerge upon the new onset of sequenced dry/wet seasons carrying new opportunities to encounter suitable hosts (Kebreab and Murdoch, 1999; López-Granados and García-Torres, 1999). Interestingly, experimentation carried out on broomrape species specialized on summer crops revealed their lower requirement for conditioning when compared with species specialized in winter annual crops highlighting the ecological adaptation of broomrape weeds to the cropping system in which they become specialized (Plakhine et al., 2009).

The metabolic activity of the seed conditioning in broomrape has been characterized in terms of patterns of respiration, synthesis and turnover of proteins, metabolism of nitrogen, carbohydrates and lipids and hormonal balance. Seed respiration patterns during conditioning indicate a strong activation of metabolism. Neither nitrogen nor lipid content change significantly during conditioning, while carbohydrate metabolism and protein synthesis seems to be crucial (Bar-Nun and Mayer, 1993, 2002; Mayer and Bar-Nun, 1994, 1997). It is well-established in autotrophic plants that abscisic acid (ABA) acts as a positive regulator of induction of seed dormancy and its maintenance and gibberelins (GAs) antagonizes with ABA, promoting dormancy release and subsequent germination (Finch-Savage and Leubner-Metzger, 2006). Accordingly, broomrape seed conditioning induces a decrease in ABA levels (Chae et al., 2004; Lechat et al., 2012) and GA synthesis (Joel et al., 1991; Zehhar et al., 2002). The reduction of ABA:GA ratio induced by stratification (conditioning) is enough to break dormancy and promote germination in dormant seeds of non-parasitic weeds but it is not enough for broomrape, which requires a further decrease in ABA levels induced by the activation of the ABA catabolic gene *PrCYP707A1* (Lechat et al., 2012). This gene remains silenced during conditioning phase and its activation occurs mediated by host-encoded germination stimulants, i.e., strigolactones, only after the conditioning phase is complete. Gain of host sensitivity in broomrape seeds at the end of the conditioning phase is mediated by demethylation of *PrCYP707A1* promoter. Besides the demethylation of *PrCYP707A1* promoter required for host-dependent *PrCYP707A1* expression, the high levels of global DNA demethylation observed at the end of conditioning period suggest that the epigenetic process occurring during the conditioning phase may be targeting other unknown molecules during conditioning. Whether the demethylation and host stimulation are independent or related processes remains to be clarified (Lechat et al., 2015).

#### Host-Induced Germination

**FIGURE 2:**
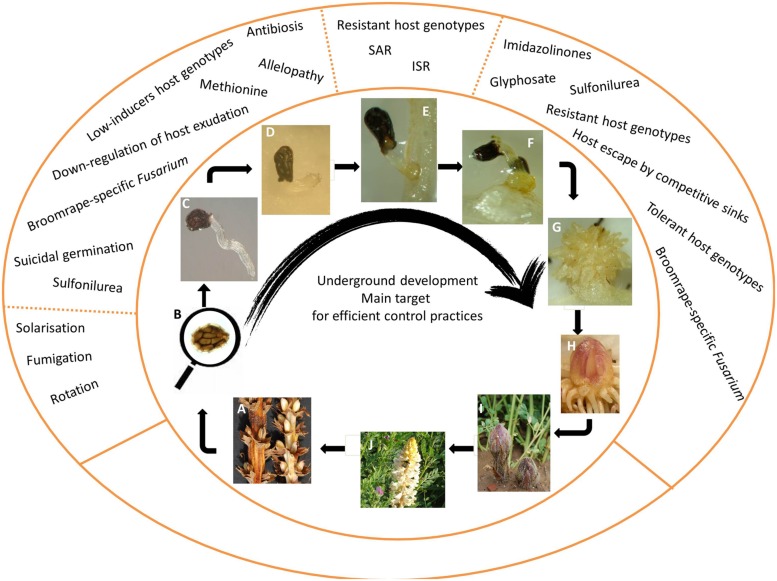
**Ilustration of broomrape life stages and mechanisms of control. (A)** Fructification and dehiscence of capsules containing mature seeds; **(B)** microscopic view of a seed (size ranging 0.2–2 mm) that undergoes sucessive dispersal, primary dormancy and annual release of secondary dormancy; **(C)** broomrape embryo does not develop morphologycaly identified cotyledons or shoot meristem and upon host-induced germination, only a radicle emerges from the seed with the function of searching and contacting the host root; **(D)** upon haustorial induction, the radicle stops elongating and a single terminal haustorium is differentiated. The first function of haustorium is as adhesion organ to host root surface mediated by a papillae cell layer; **(E)** adhesion to the root 3 days after germination induction; **(F)** upon vascular connection with the host, broomape initiates the development of the tubercle, the broomrape storage organ for host-derived nutrients. A swelling of the host root at the penetration point is also observed due the parasitic stimulation of host tissue proliferation; **(G)** tubercle develops a crown of adventicious roots; **(H)** tubercle differentiates apical shoot meristem (single shoot meristem for Orobanche species and several shoot meristems for Phelipanche species); **(I)** the underground shoot eventually emerges through the root surface; **(J)** flowering and pollination occur. Some broomrape species are outcrossers while others are self-pollinating. Reviewed in Joel et al. (2007).

The timing of germination is the most crucial event that obligated parasitic plants face along their life cycle (**Figure 2C**). Their absolute dependence on host-derived nutritive resources for successful seedling establishment and consequent growth makes necessary the synchronization of parasitic germination with the growth of its host. In order to achieve such synchrony they evolved mechanisms that release seed from dormancy triggering germination upon detection of specific molecules contained in host root exudates (Vaucher, 1823).

Broomrape species display high diversity with regard to their host range. Host specificity in broomrape species is usually indirectly related to the predictability of nutritive resources. The predictability of establishment on perennial hosts is high and therefore wild broomrape species feeding off perennial plants have narrow host ranges. On the contrary, weedy broomrape species are usually generalists attacking annual crops (Schneeweiss, 2007). The first mechanism involved in host specialization is displayed during broomrape germination and is mediated by the broomrape recognition of host root exudates in a species-specific manner. For each broomrape-crop association, broomrape germination potential is defined by the combination of both, the stimulatory capability of crop root exudates and the sensitivity of parasitic receptors to recognize specific forms of germination-inducing factors (Fernández-Aparicio et al., 2008a, 2009b, 2011).

The stimulatory capability of crop root exudates is defined by the qualitative and quantitative content of germination-inducing factors and varies across crop species and cultivars. In addition it also varies considerably in crops growing under different physiological status, growth stages and growing seasons, allowing broomrape to synchronize its germination with physiologically suitable hosts (López-Granados and García-Torres, 1996; Yoneyama et al., 2007a,b; Fernández-Aparicio et al., 2009b, 2014; Xie et al., 2010). Several classes of germination stimulants have been identified in root exudates such as strigolactones (Xie et al., 2010), peagol and peagoldione (Evidente et al., 2009), peapolyphenols A–C (Evidente et al., 2010), soyasapogenol B, *trans*-22-dehydrocampesterol (Evidente et al., 2011), dehydrocostus lactone (Joel et al., 2011), or isothyocyanates (Auger et al., 2012). The best studied group of germination-inducing factors are strigolactones, a group of terpenoid lactones. They are exuded by the crop to the rhizosphere under nutrient deficient conditions in order to promote symbiotic interactions (Akiyama et al., 2005). Parasitic plants eavesdrop the plant-to-symbiont communication to sense their hosts and germinate (Xie et al., 2010). Besides their role as extraorganismal signaling, recent research is uncovering new functions for strigolactones as plant hormone controlling crop development in response to the environment (Gomez-Roldan et al., 2008; Umehara et al., 2008). Post-germination development in broomrape could be probably regulated by their own broomrape-encoded strigolactones as it occurs in the close related parasite *Striga hermonthica* or in non-parasitic plants (Liu et al., 2014; Das et al., 2015). Although analytical chemistry methods have failed to detect strigolactones in parasitic plants (Liu et al., 2014), transcriptome sequencing reveals that all known strigolactone genes, both synthesis and perception are present in broomrapes with apparently full-length proteins (Péron et al., 2012; Das et al., 2015). The presence of strigolactone biosynthetic system in broomrapes raises the question on how the parasite performs diversified stimulant recognition in order to set the timing of germination. How broomrapes make the distinction not only between host-derived and their own-encoded strigolactones but also how they sense diversified strigolactone profiles in root exudates across species correlated with host ranges. Gene expression analysis could be indicating that parasitic plants down-regulate their synthesis of strigolactones at the end of conditioning period, and perhaps the creation of that internal deficit for broomrape-encoded strigolactones contributes to the broomrape sensitivity for external, host-derived strigolactones at the time of host detection (Das et al., 2015). In addition, the parasitic-specific receptor KAI2d that enables host detection in broomrapes has recently been identified. Multiple *KAI2d* genes across broomrape species genomes may allow diversified recognition of root exudates corresponding with suitable hosts (Conn et al., 2015). Additional mechanisms that could contribute to the selective action of host-derived strigolactones in broomrape germination could be (1) variations of molecular structure between host-derived and parasite-encoded strigolactones conferring different specificity for different biological functions or (2) different spatial localization inside the broomrape seed for functions of strigolactone detection and strigolactone synthesis (Das et al., 2015).

The requirement for germination-inducing factors in order to break dormancy in parasitic seeds are bypassed by ethylene or cytokinins (which promotes ethylene biosynthesis) in *Striga* sp. a close related parasitic weed genus, but these hormones are ineffective in promoting germination of broomrape weeds (Lieberman, 1979; Logan and Stewart, 1995; Berner et al., 1999; Joel, 2000; Toh et al., 2012). GA acts positively on germination in dormant non-parasitic species by counteracting ABA (Seo et al., 2009). However, exogenous application of GA alone is not sufficient to promote broomrape germination (Takeuchi et al., 1995; Chae et al., 2004) and strigolactone-mediated ABA catabolism in conditioned seeds is required to trigger germination (Lechat et al., 2012). A better understanding in the roles of major hormones in the process of broomrape germination would facilitate the design of feasible control strategies based on either inhibition of broomrape germination during crop cultivation or promotion of suicidal germination in the absence of the crop. In order to increase their applicability in low-input crops, the development of synthetic analogs of hormones would constitute a cheap alternative to natural bioregulators for seed bank control of weeds in general and parasitic weeds in particular.

#### Radicle Elongation and Haustorium Differentiation

The embryos in broomrapes have not morphologically identified cotyledons or shoot meristems and upon germination, only a radicle emerges through the seed coat with the only function of reaching and invading the host. The broomrape radicle shows no gravitropism and grows toward the host as a result of cell elongation. It has no root cap and does not develop procambium or conductive tissues (Joel and Losner-Goshen, 1994). Being deprived of the initiation of autotrophic mode of life, the growth of broomrape seedling toward the host is only sustained by water absorption and remobilization of reserve nutrients from the seed perisperm and endosperm (Joel, 2000; Joel et al., 2012). The seedling absorbs water both from the soil and from the seed endothelium, the later ensuring radicle development even in dry soil (Joel et al., 2012). The maximum radicle elongation is limited (1–5 mm) and its viability in the absence of host connection only last a few days after germination has been triggered (Veronesi et al., 2007). This spatial/temporal frame defines the maximum host-reaching distance for successful broomrape parasitism. Upon host detection, the broomrape radicle stops elongating and terminal haustorium is differentiated as an anchoring device. In this process, cellular expansion of the root meristem is redirected from longitudinal to radial and the root apex changes its form from conical to spherical. The external cell layer at the root tip differentiates into a papillate cell layer forming an adhesion epithelium (**Figure 2D**). The papillae form a crown around the apical cells that remain non-papillate but later will become intrusive cells with an essential function in the penetration process. This surface is covered by carbohydrate secretion that sticks the haustorium to the host surface. This structure is described as the external anchorage device of the pre-penetrated haustorium to the host surface (Joel and Losner-Goshen, 1994).

Close related parasitic plants of Orobanchaceae such as *Striga* and *Triphysaria* use host derived phenolic derivatives to induce haustorium differentiation (Riopel and Timko, 1995; Albrecht et al., 1999; Bandaranayake and Yoder, 2013). Hydrogen peroxide generated by parasitic radicles activates host peroxidases that catalyze the conversion of host cell walls into haustorium-inducing quinones (Keyes et al., 2000, 2007). Haustorium-inducing factors are structurally similar to allelopathic phytotoxins and gene expression of parasitic radicles exposed to haustorium-inducing factors is similar to that after radicle is exposed to phytotoxins (Tomilov et al., 2006). Parasitic plants probably evolved to recruit plant defense molecules as host recognition cues (Atsatt, 1977; Matvienko et al., 2001; Bandaranayake and Yoder, 2013). In broomrape species, the chemistry of host recognition for haustorium initiation remains uncharacterized. They have been traditionally considered the exception in parasitic Orobanchaceae that do not require host factors for haustorium initiation (Joel and Losner-Goshen, 1994; Bandaranayake and Yoder, 2013). However, results recently arisen from a molecule screening identified phytotoxins that induce the development of anchoring device in broomrape radicles (Cimmino et al., 2014, 2015). The significance of this structure in broomrape parasitism requires further investigation. A better understanding of the biochemistry of host recognition in broomrape will facilitate the generation of control strategies targeting the haustorium development.

### Host Invasion

After host adhesion to host root surface the haustorium develops its invasive function of penetrating the host root (**Figure 2E**). The apical cells in the radicle apex develop into intrusive cells, which successively invade host root cortex, endodermis, and the central cylinder. During the host penetration process, broomrape does not dissolve the host cells in its way toward vascular cylinder. Mechanical force exerted by the haustorium development toward host vascular cylinder combined with enzymatic secretion promotes the separation of host cells without their lysis (Privat, 1960; Ben-Hod et al., 1993; Sholmer-Ilan, 1993; Singh and Singh, 1993; Antonova and Ter Borg, 1996; Bar-Nun et al., 1996; Losner-Goshen et al., 1998; Veronesi et al., 2005). Although broomrape pre-vascular connections benefits from host nutrients, the growth of broomrape in its way toward vascular cylinder is mainly sustained by consumption of seed reserves (Aber et al., 1983; Joel and Losner-Goshen, 1994; Joel, 2000). If the vascular connection is not successfully performed in few days the parasitic seedling dies of inanition and therefore quick invasion of the host is of advantage to avoid loss of viability. When resistant crops impose barriers to stop the parasitic development at this stage, broomrape exhausts and parasitism is quickly aborted. The chemical characteristics of the barriers of resistance to broomrape penetration have been extensively studied in Fabaceae crops (Pérez-de-Luque et al., 2009) and are reviewed in this article in Section “Resistant Crops to Broomrape Invasion.”

### Establishment of Vascular Continuity and Parasitic Sink

Shortly after host penetration and connection, the parasite begins its heterotrophic growth at the expense of host resources. Broomrape acts as a strong sink, depriving the host from water, mineral, and organic nutrients with the consequent negative impact on the growth of the host plant (Manschadi et al., 1996; Hibberd et al., 1998; Joel, 2000; Abbes et al., 2009). Few days after host vascular connection, the part of the broomrape seedling that remains outside the host root develops into a storage organ called tubercle. As the tubercle matures a crown of adventitious roots will emerge from this tubercle carrying capacity of developing lateral haustorial connections. Underground shoots will also develop from the tubercles that will eventually emerge through the soil surface leading into the development of reproductive organs (**Figures 2F–J**).

The transfer of nutrients from host to broomrape is performed through a continuous vascular system at the host-parasite interface. A continuous phloem system between broomrape and its host has been microscopically observed at the terminal haustoria. Sieve elements of both organisms are already interconnected by interspecific sieve pores at early stages of parasitism. It allows the parasite to quickly start tapping carbohydrates, amino acids, and organic acids from its host (Dörr and Kollmann, 1995; Nandula et al., 2000; Abbes et al., 2009). Although host phloem supplies the majority of nutrients including minerals, open xylem connections developed at the host-parasite interface allow additional mineral and water flow toward the parasite (Abbes et al., 2009; Westwood, 2013). These connections are probably developed from simultaneous differentiation of adjacent host and parasite cells to xylem elements (Dörr, 1997). The differentiation of xylem elements in the parasite are under the control of polar auxin transport (Harb et al., 2004; Bar-Nun et al., 2008). During the grafting between host and parasite, broomrape assumes the role of a root, orientating vascular tissues from the host shoot into itself (Bar-Nun et al., 2008). Acquisition of water is driven by a lower water potential in broomrape tissues (Ehleringer and Marshall, 1995). This is maintained by accumulation of solutes mainly potassium at higher concentrations than in the corresponding host tissues (Abbes et al., 2009). Regarding carbon assimilation broomrape takes it from the host phloem mainly in the form of sucrose (Aber et al., 1983; Hibberd et al., 1999). Once in the parasite system, sucrose is not accumulated but metabolized to other compounds. Cleavage of sucrose into glucose and fructose doubles the osmotic potential of the parasite. Sucrose is also metabolized to starch that is accumulated in the broomrape storage organ, the tubercle (Abbes et al., 2009; Draie et al., 2011).

Nitrogen metabolism remains largely unknown in broomrape. Activity of some nitrogen assimilating enzymes has been reported low in broomrapes. Nitrate reductase is not detectable (Lee and Stewart, 1978) and activity of glutamine synthetase is very low (McNally et al., 1983). Broomrape tubercles accumulate host-derived nitrogen in the form of either arginine or in the arginine and aspartate pair (Nandula et al., 2000; Abbes et al., 2009). Besides arginine and aspartate, other major forms of amino acids translocate from the host phloem but they are rapidly utilized by broomrape. Maintenance of relative low levels of those amino acids in tubercles either by low levels of synthetase activities (McNally et al., 1983) and or their rapid turnover of host-derived amino acids, establishes a decreasing concentration gradient that favors the unloading of amino acids into the parasite (Abbes et al., 2009). Despite the reports of broomrape inefficient machinery for nitrogen assimilation and broomrape dependence for host-derived organic forms of nitrogen demonstrated by the fact that broomrape growth is arrested when feeding on host cultivars with decreased amino acid-phloem levels (Abbes et al., 2009), inhibition of enzymes at the top of amino-acid biosynthetic pathway by means of either direct inhibitory action of herbicides (Gressel, 2009) or by feedback inhibition induced by amino-acid end-products (Vurro et al., 2006) are able to kill broomrape. This seems to indicate contribution of amino acid synthesis in broomrape mediated by broomrape-encoded enzymes although their identification and characterization remain unknown (Gressel, 2009; Eizenberg et al., 2012).

Understanding the key processes of host recognition, haustorium development and maturation and metabolic regulation of the parasitic sink allow virulence predictions and the design and implementation of highly calibrated, feasible, and durable control strategies leading to the arrest of broomrape parasitism minimizing simultaneously environmental impact and yield losses.

## Control Strategies Targeting Underground Broomrape Stages

Successful broomrape control should target the underground broomrapes at their earlier life stages, prior attachment or as soon as it attach to the host, because of their highest vulnerability at those stages and the avoidance of yield loss in the current crop. The majority of strategies aimed to manage autotrophic weeds do not necessarily work for broomrapes and those that provide a degree of success for broomrape need to be optimized for each broomrape-crop species combination, local environmental conditions and broomrape population. Due to the high broomrape fecundity, long seed viability and for some weedy broomrape species, broad host range, the seed bank is easily replenished and long lasting. Therefore an integrated and sustained management strategy composed by several control methods acting at different broomrape life stages is highly recommended to keep away the broomrape weed problem in a durable manner (Kebreab and Murdoch, 2001). The following sections and **Table 1** review the major feasible control measures for broomrape control.

**Table 1:** Methods for *Orobanche* and *Phelipanche* spp. control.

Technique	Broomrape stage targeted	Feasibility	Drawbacks or side effects
**Cultural control**					
Rotation including	Trap crops Catch crops Allelopathic crops		Seed germination Pre-attached seedling Young attachments	To avoid seed bank replenishing the rotation should avoid • Susceptible crops • Broomrape-susceptible non-parasitic weeds	• Miscalculation of optimal harvesting time for catch crops leads to broomrape multiplication • Avoidance of susceptible crops that in most cases are the most economically advantageous • Slow process requiring long term management perspective • Risk of widen broomrape host range

Intercropping susceptible crops with allelopathic species			Seed germination Pre-attached seedling	• Requires appropriate selection of sowing density and non-host component for each broomrape species • Requires greater management skills • Once optimized, rather easy to set up	• Yield uncertainty • Partial effect within a growing season

Fertilization	Nitrogen		Direct effect on seed germination and pre-attached stages	• Urea and ammonium but not nitrate forms inhibit broomrape seed germination and radicle elongation	• Environmental pollution • Potentially inappropriate Nitrogen supply for the crop species • Lower levels of symbiotic interactions in legume crops
			Indirect effect on seed bank: negative regulation of host synthesis and exudation of germination-inducing factors	• Nitrogen regulates host exudation of germination-inducing factors in some species such as sorghum, lettuce, vetch, and wheat but not in important broomrape hosts such as clover, alfalfa, and tomato
	
	Phosphorus		Indirect effect on seed bank: negative regulation of host synthesis and exudation of germination-inducing factors	• At low or high pH the P solubility is reduced • Requires correct placement of the fertilizer both for easy access to crop root (P is immobile) and to avoid toxic effect to the crop seed (DAP-containing fertilizers produce free ammonia)	• Environmental pollution • Limited global resources. • Lower levels of symbiotic interactions in crops

Delayed sowing			Ungerminated seed bank (lower stimulatory capability in root exudates of mature crops growing late in the season) Young attachments (lower parasitic sink strength competing with rapid seed-filling stage of host fruits)	• Requires proper timing to obtain the best balance between inhibition of parasitic sink strength and crop productivity	• Shorter growing cycles not only reduces the amount of biomass partitioned to the parasite but also the overall crop productivity

**Physical control**					
Solarization			Ungerminated seed bank	• Requires high solar radiation	• Expensive • Plastic waste • For intercropping periods

**Chemical control**					
Soil fumigation	Methyl bromide		Ungerminated seed bank	• Banned by international agreement	• Environmental pollution • Expensive • Labor intensive
	
	Metham sodium Dazomet 1,3-dicloropropene		Ungerminated seed bank	• Tested as substitutes of methyl bromide but they are more expensive and less effective	• Environmental pollution • Expensive • Many active ingredients removed from the market for their unacceptable environmental profile under recent legislation • Labor intensive

Soil herbigation	Synthetic herbicides	Sulfonylurea	Seed germination Radicle elongation Young attachments	• Requires proper timing and application technology. • Requires a crop tolerant to sulfonylurea residues	• Environmental pollution. • High risk of appearance of sulfonylurea-resistance races.
	
	Inducers of suicidal broomrape germination	Synthetic analogs of strigolactones Fluridone GA agonists	Ungerminated seed bank	• Requires proper timing, application technology • Should be applied in absence of susceptible crops in order to avoid further increase of parasitism	• Though suicidal germination has been proved successful for *Striga* weed control in USA, it has not reached acceptable levels of seed bank reduction in broomrape
	
	Broomrape-specific phytotoxic amino acids	Methionine Lysine	Seed germination Radicle elongation Crop invasion	• Green and non-toxic • Requires proper timing, application technology, and tolerant crops • Commercially available at large scale as animal feed supplements • Mixed applications of two inhibitory amino acids can bring synergistic effect on weed control and reduced risk of resistance breakdown adaptation	• Soil pH alteration • Difficult application at the required broomrape toxic concentration (5 mM) • Technique under experimental development
	
	Toxins from microbial origin	Deoxynivalenol Diacetoxyscirpenol Fusarenon X HT-2 toxin Neosolaniol Nivalenol Roridin A T-2 toxin Verrucarrins A, B and M	Seed germination Radicle elongation	• Molecules of biological origin are biodegradable in comparison with synthetic herbicides • Requires proper timing and application technology • Requires drip-irrigation system for site-specific weed management and avoidance of toxin spread • Broomrape-specific toxins are preferred	• Lack of broomrape selectivity • Lack of knowledge on the physiological mechanism responsible for inhibitory action • Lack of knowledge on the persistence in the soil • Technique under experimental development • Lack of large scale production

Foliar application of systemic herbicides		Glyphosate Imidazolinones Sulfonylurea	Attached parasites (herbicide reaches the parasite via the haustorium)	• Requires proper timing and application technology • Requires crops with herbicide-resistance not based on metabolic degradation or inactivation • Where herbicide-resistance varieties are not available within a crop, repeated application at low doses are effective for young parasitic stages • Exact knowledge of the underground phenology related with thermal time is required to predict the best application timing	• Marginal crop selectivity • Environmental pollution • Injury of reproductive meristems in some crops • Sublethal concentrations of glyphosate suppress phytoalexin-based immunity in legumes toward pathogens • GM crop paradigm

**Biological control**					
Broomrape-specific pests and pathogens Fungi and bacteria	Solid formulation	Wheat, corn or rice seeds Granular formulation (microbial agent+nutrients)	All stages from un-germinated seed bank to broomrape inflorescences	• Risk of low uniform distribution and control efficiency • Control efficiency can be increased by “multiple pathogen strategy” • Control efficiency can be increased with abiotic elicitors of resistance, e.g., BTH or sublethal herbicidal doses	• Allows only one application (at planting) • Moisture is limiting factor • Low uniform distribution and control efficiency • Regularity of the result can hardly be ascertained because of biotic interactions prevailing in the local conditions

Wild forms of fungal and bacterial pathogens	Microbigation		All stages from un-germinated seed bank to broomrape inflorescences	• Requires drip-irrigation system for site-specific weed management • Repeated applications of *Fusarium* suspensions provide moisture and optimization of concentration and timing	• Still under scientific evaluation. PGPR area to which this technique is related remains largely unexplored

Enhanced-virulence pathogens	Hypervirulence transgenes Pathogens overproducing and excreting phytotoxic amino acids	Enzymes that overcome broomrape defense mechanisms Broomrape-specific toxins	All stages from un-germinated seed bank to broomrape inflorescences	• Increased broomrape kill rate in comparison with their wild forms • Enhanced virulence should not alter specificity for broomrape weed • Spread of transgenic organisms could be avoided with the selection of asporogenic mutants	• Still under scientific evaluation. Techniques remain largely unexplored

Insects	*Phytomyza orobanchia*		Avoids seed bank replenishing	• Broomrape-specific • Efficiency can be lowered by several agricultural practices and parasitoids • Efficient control requires multiple applications over several years • No artificial diet for rearing is available • Easiness to add to other agricultural controlling practices	• Low impact in a short term seed bank management • No impact in the productivity of the current crop

Symbiotic organisms	*Rhizobium leguminosarum Glomus mosseae*		Seed germination Radicle growth Crop invasion	• Requires optimal control of fertilization	• Strategy in its early developmental stage

**Host resistance**					
Breeding for natural resistance	Broomrape-resistant varieties		Seed germination Host attachment Crop invasion	• Low cost of implementation once developed • Environmental compatible • Varieties with pyramiding gene resistance are preferred to avoid resistance breakdown by the parasite • Resistant varieties should be used in an integrated management strategy to avoid resistance breakdown by the parasite	• Not available for all crops • Risk of emergence of new virulent races
	
	Broomrape-tolerant varieties		Parasites attached to the host	• Feasible • Environmental compatible	• Not available for all crops
	
	Systemic herbicide-resistant varieties		Young parasitic seedling connected to host vascular system	• Feasible if herbicide resistance is not based on metabolic degradation or inactivation • Requires proper timing and application technology	• Not available for all crops • Risk of emergence of herbicide-resistant broomrape populations

Mutagenised crops	EMS-mutagenesis Fast-neutron-mutagenesis		Seed germination Host attachment Crop invasion	• Low cost of farm implementation of resistant varieties • Environmental compatible	• Risk of emergence of new virulent races

Transgenic resistance	Transgenes encoding for toxic products to broomrape		Young broomrapes	• Low cost of farm implementation of resistant varieties • Better results are obtained when using broomrape-responsive gene promoters	• Technique under experimental development • Public acceptance in some countries
	
	RNA interference		Young attached parasites (broomrape-specific RNAi reaches via the haustorium)	• Low cost • Environmental compatible • Requires previous identification of parasitic genes essential for broomrape virulence and metabolism • Resistant varieties should be used in an integrated management strategy to avoid resistance breakdown by the parasite	• Genetic redundancy may dilute silencing effect • Technique under experimental development
	
	Transgenic herbicide resistance		Attached parasites (herbicide reaches the parasite via the haustorium)	• Herbicide-resistance mechanism must be based in mechanism other than metabolic degradation or inactivation of herbicide by the transgenic crop • Low herbicide doses are required to kill the young parasite	• Food safety issues • Herbicide resistant-gene transfer to wild plants • Public acceptance in some countries

### Management Strategies to Protect Crops from Detection by Broomrape Seeds

Due to the small size of the seeds and their inability to develop autotrophy, the establishment probability of broomrape seedlings is very low. Broomrapes counteract the high risk of failure in establishment on a host with highly evolved mechanisms of survival. Broomrape high fecundity, with thousands of seeds released per broomrape plant (**Figures 2A,B**), multiplies the chances of the next generation to encounter a host and achieve successful parasitism (Parker and Riches, 1993). In addition long lived seed banks under physiological dormancy ensure that germination will occur when a suitable host in its correct stage of development is present nearby (Rubiales et al., 2009b). Preventing the movement of parasitic seeds from infested to non-infested agricultural fields, by contaminated machinery or seed lots, is crucial (Panetta and Lawes, 2005). Once a field is infested, controlling the broomrape seed bank is very difficult due to its high resilience. A variety of methods have been developed to specifically neutralize broomrape pre-attached development though the majority of them are not commercially implemented because they are still at the stage of development or have not proved enough efficiency or applicability for large scale crops. These methods can be classified as cultural and physical, chemical, biological control, and use of host resistance (Rubiales et al., 2009b).

#### Cultural and Physical Control Practices

Fertilization can induce soil suppressiveness to initiation of broomrape parasitism. Application of phosphate or nitrogen to deficient soil reduces broomrape parasitism on clover and tomato (Southwood, 1971; Jain and Foy, 1992). Nutrients influence the crop-parasite pre-attached interaction in several ways. Direct toxic effects by urea and ammonium but not nitrate forms inhibit broomrape seed germination and radicle elongation (Jain and Foy, 1992; Abu-Irmaileh, 1994; van Hezewijk and Verkleij, 1996; Westwood and Foy, 1999). Accumulation of ammonium can be toxic to plants and its detoxification occurs via incorporation into organic compounds. The activity of glutamine synthetase in broomrape is very low and therefore carries a reduced broomrape ability to detoxify ammonium. Urea has no detrimental effects in plants but it is toxic to broomrape pre-attached stages probably exercised via ammonium after broomrape urease hydrolyses urea into ammonium. Nitrate is not toxic to broomrape as it lacks the ability to convert nitrate into ammonium (van Hezewijk and Verkleij, 1996). In addition to the toxic effects on broomrape seed and seedling, fertilization can protect crops from broomrape parasitism by means of down-regulating the crop synthesis and exudation of strigolactones, the most potent germination-inducing factors for broomrape. Phosphorous and nitrogen have been described to down regulate strigolactones exudation in some crop species (Yoneyama et al., 2007a,b, 2012). This effect may not be applicable to those broomrape species with preference for classes of germination-inducing factors other than strigolactones (Joel et al., 2011; Auger et al., 2012).

Intercropping systems cultivate simultaneously more than one species in close association to take agronomic advantage of biodiversity, competition, and complementarity between them. For broomrape control, this system seeks the simultaneous cultivation of susceptible host species with inhibitory species of broomrape parasitism. The release of phytochemicals by the roots of the allelopathic component in the intercrop inhibits the broomrape germination and/or radicle elongation toward the host component. Successful reduction of broomrape parasitism in the current crop is obtained by intercropping host species with inhibitory species of cereals, fenugreek, or berseem clover (Fernández-Aparicio et al., 2007, 2008b, 2010a). Careful selection of the non-host component in the intercrop is, however, required as some plant species can act as non-host facilitators and therefore increase the severity of broomrape infection in the host component (Gibot-Leclerc et al., 2013).

A rotation decreasing the frequency of host cultivation is one of the main ways that farmers deal with the broomrape-related problem. This prevents broomrape parasitism from taking place, maintaining the seed bank dormant and reducing the rate of seed bank replenishing. However, it is a long-term strategy due to the long viability of seed bank (Rubiales et al., 2009b), which requires at least a nine-course rotation in order to prevent broomrape seed bank increases (Grenz et al., 2005). Its efficacy for broomrape cultural control can be increased if the farmer includes trap and/or catch crops as components in the rotation (Rubiales et al., 2009b). The concept of trap crops refers to the cultivation of crop species whose root exudates exhibit high germination-inducing activity on broomrape seeds, but these species do not become infected because they are resistant to later stages of the parasitic process indirectly leading to the killing of the young broomrape seedlings due to the lack of proper host. The inductor potential of root exudates from a given species varies with the broomrape considered. Each broomrape species show specificity not only for root exudates in order to germinate but also for host species to invade and feed on, being the germination-stimulatory range usually broader than the actual host range (Fernández-Aparicio et al., 2009b). Potential trap crops have been suggested for broomrape weeds (Parker and Riches, 1993). For instance, root exudates of field pea induces high germination of the very destructive broomrape species *O. crenata, O. foetida, O. minor*, and *P. aegyptiaca*, however, it only becomes infected by *O. crenata* therefore pea may theoretically be a good trap crop against *O. foetida, O. minor*, and *P. aegyptiaca* but not for *O. crenata* infested field (Fernández-Aparicio and Rubiales, 2012). Many other interesting examples of trap crops emerged from a root exudates screening of important crops (Fernández-Aparicio et al., 2009b). The second possibility to increase rotation efficacy for broomrape control is to include catch crops, which are crops that also induce high broomrape germination but they are not resistant to it. On the contrary, they must be highly susceptible, as the farmer is the one with the role of stopping the parasitic process by harvesting the catch crop as a green vegetable before the parasite emerges. For instance, tori (*Brassica campestris* var. *toria*) when managed properly as a catch crop can result in up to a 30% reduction in the size of broomrape seed bank (Acharya et al., 2002).

Soil management affects the success of broomrape seeds in becoming established on the host and then the longevity of broomrape seed bank. Minimum tillage reduces the amount of viable seeds incorporated in the soil and then their capacity to reach the crop root system (Ghersa and Martinez-Ghersa, 2000; López-Bellido et al., 2009). The opposite agricultural practice deep-plowing, has been suggested to bring seeds of parasitic weeds to a depth with less oxygen availability and therefore a reduction in its germination capacity (Van Delft et al., 2000). According with pot experiments carried out in the tomato-*P. aegyptiaca* system, deep-plowing bringing the seeds to depth ≥ 12 cm will strongly reduce broomrape infection severity in terms of number of parasites, total parasitic biomass, delayed broomrape emergence and prevention of flower initiation and seed set (Eizenberg et al., 2007).

Solarization is a thermal soil disinfestation method that shows high efficiency reducing the viability of the broomrape seed bank along with other harmful organisms to crops such as plant-parasitic nematodes, disease causing microorganisms and non-parasitic weeds. This method consists in heating the soil through sun energy achieving temperatures above 45°C, by covering a wet soil with transparent polyethylene sheets for a period of 4–8 weeks during the warmest season (Katan, 1981; Mauro et al., 2015). Many beneficial organisms are either able to survive the solarization treatment or able to recolonize solarized soil (Sauerborn et al., 1989; Mauromicale et al., 2001). Not all areas infested by broomrape are suitable for efficient solarization. Hot air temperature and clear skies are required during the solarization period. Its high cost per surface unit makes this method not readily applicable at large scale (Joel, 2000). In addition, this technique generates a considerable amount of plastic waste but the emergence of new materials at low-cost, of biological origin and biodegradable may in the future reduce earth pollution with plastic debris derived from agriculture practices (Fernandez and Ingber, 2013).

#### Chemical Control of Seed Bank

Soil fumigation with methyl bromide has been proved one of the most effective methods to eradicate broomrape seed bank, but this chemical has been banned from use due to its toxic effects on the environment (Joel, 2000; Hershenhorn et al., 2009). Use of other soil sterilants such as metham sodium, dazomet, and 1,3-dichloropropene have shown different degrees of efficacy but their high cost, complex application and negative environmental effects have prevented their widespread use by farmers (Foy et al., 1989; Goldwasser et al., 1995; Hershenhorn et al., 2009) or conducted to the withdrawal of authorization, at least in some countries.

Soil herbigation (saturating the soil with herbicides such as sulfonylureas) effectively controls preattached stages of broomrapes (Hershenhorn et al., 2009) but is hardly compatible with other agricultural cropping practices as detrimental for many crop seedlings for several weeks or months. *In vitro* treatments of a large range of sulfonylurea herbicides inhibit broomrape germination and radicle elongation (Hershenhorn et al., 1998; Plakhine et al., 2001). Incorporation of sulfosulfuron and rimsulfuron directly to the soil provides successful control of preattached stages of broomrape weeds (Eizenberg et al., 2012).

Natural pesticides derived of microbial and plant origin are considered to be less harmful because they usually biodegrade quicker, resulting in less pollution-related problems. Several toxins have been identified with inhibitory activity on broomrape parasitism by interfering with broomrape germination and radicle elongation (Vurro et al., 2009; Fernández-Aparicio et al., 2013; Cimmino et al., 2014). Special interest arises from those metabolites with a favorable pattern of broomrape-specific effect (e.g., tenuazonic acid) and no described side-effect to other biosystems (Vurro et al., 2009). However, the efficacy of these molecules has been proved only in laboratory essays. Until now, difficulties of purification at industrial scale have hampered the field experimentation with such metabolites (Vurro et al., 2009) despite their interesting potential.

Certain amino acids strongly inhibit the early development of broomrape without phytotoxic effects in the host (Vurro et al., 2006). The amino acid approach to control weeds is inspired on the concept of frenching disease where amino acid end-product inhibits the activity of a controlling enzyme in the amino acid biosynthesis pathway (Vurro et al., 2006, 2009; Sands and Pilgeram, 2009). The effectiveness of amino acids as broomrape inhibitors has not been proved in real field conditions but field application of amino acids has been effective to manage other parasites such as plant-parasitic nematodes (Zhang et al., 2010). Among the amino acids producing the highest and most consistent inhibitory effects on broomrape germination and radicle elongation, some, such as methionine are being produced in large commercial scale as animal feed supplements. The use of those amino acids as pesticide is classified by the United States Environmental Protection Agency as innocuous to public and environment health (USEPA, 2004). Based on those conditions, methionine has the potential to be used as broomrape herbicide but it needs to be confirmed and its application adjusted to real field conditions.

Synthetic analogs of growth regulators can be successfully used to reduce parasitism by hampering the synchronization of the parasitic seed bank with the growth of the host. Inhibition of seed conditioning and subsequent germination mediated by inhibitors of GA synthesis reduces the receptivity of broomrape seeds to germination-inducing factors. In addition, inhibitors of ABA catabolism inhibit the germination-triggering effect of host-derived strigolactones. For example, soil application of uniconazole, a triazole that is commercially used for growth regulation has proved to reduce parasitism by inhibiting seed conditioning and subsequent germination (Joel, 2000; Zehhar et al., 2002; Song et al., 2005; Lechat et al., 2012).

Promotion of suicidal germination is the technique used to induce broomrape germination with synthetic molecules in the absence of a host to which broomrape can attach, a technique lethal for the parasite as the broomrape seedling is unable to acquire autotrophy. Therefore, it may be possible to achieve broomrape control by fooling the parasite with the delivering to the soil of synthetic analogs of the original host-derived germination-inducing factors such as strigolactones (Johnson et al., 1976). Direct application of strigolactones to the soil has been the subject of intense research. The first attempts to deplete parasitic weed seed bank was made by Johnson et al. (1976) by using the synthetic strigolactone analog GR7. However, instability of this compound, particularly at pH > 7.5, and lack of optimal formulations rendered this technique not applicable (Saghir, 1986; Babiker et al., 1987, 1988). Recent advances in this research area has led to new, more stable strigolactone analogs and optimization of field application protocols and formulations (Bhattacharya et al., 2009; Zwanenburg et al., 2009; Mwakaboko and Zwanenburg, 2011). Another strategy to induce suicidal germination of broomrape seed bank could be the use of gibberellin agonists. They elicit GA-like germination activity in dormant seeds of several autotrophic plant species (Suttle and Schreiner, 1982; Metzger, 1983), constituting a cheap alternative to natural bioregulators for weed seed bank control (Suttle, 1983). When they are applied *in vitro* to seeds of *P. ramosa* and *O. minor*, they bypass the effect of germination-inducing factors, promoting broomrape germination in absence of host or any germination stimulant (Cala et al., 2015).

Once broomrape germination has occurred, chemicals that reduce the growth of broomrape radicle reduce the chances of reaching the host and therefore parasitism. A novel metabolite, ryecyanatine-A recently isolated from rye (*Secale cereale* L.), presents potential for broomrape control by promoting rapid cessation of broomrape radicle growth and therefore inhibiting its ability to reach the host. In addition it promotes the development of a layer of papillae at the radicle apex in the absence of host contact, morphology that resembles the attachment organ (Joel and Losner-Goshen, 1994; Cimmino et al., 2015). Because the haustorial organ in broomrape radicle is terminal and its growth is not resumed unless it can immediately penetrate the host, cessation of radicle elongation and haustorial induction in the absence of a host is lethal to the parasite. Other interesting molecules that hamper the ability of broomrape radicle to reach the host have been recently discovered from different microbial and plant origins (Fernández-Aparicio et al., 2013; Cimmino et al., 2014).

#### Biological Control of Seed Bank with Living Organisms

Biological control of broomrape is based on the use of living organisms either by killing seed bank or interfering with its host-recognition ability. Assessment of pathogenicity or damages toward non-target plants has to be carefully assessed in order to avoid environmental risks. The efficient action of the biological control agent will depend on its ability to remain active over a large range of ecological conditions (Aly, 2007).

##### Phytophagous insects to prevent the build-up of broomrape seed bank

More than 40 insect herbivores from 22 families have been collected on broomrape plants but a majority of them are polyphagous without any specificity for broomrape species being some of them serious pests of important crops (Klein and Kroschel, 2002). *Phytomyza orobanchia* is reported to be broomrape-specific and its main action as biocontrol agent is by reduction of broomrape reproductive activity due to their feeding activity on ovules and young seeds. Often secondary infections by fungi cause early death of broomrape shoots or limit the development of flowers and ovules (Klein and Kroschel, 2002). The capacity of *P. orobanchia* to reduce broomrape populations is limited by cultural practices and antagonists (Klein and Kroschel, 2002; Aly, 2007).

##### Mycoherbicides attacking broomrape seeds and radicles

The broomrape seed bank efficiency to initiate parasitism can be reduced by incorporation to the soil of several pathogens able to infect preattached broomrape stages such as *Fusarium* sp. or *Ulocladium botrytis* (Müller-Stöver, 2001; Boari and Vurro, 2004; Dor and Hershenhorn, 2009). Like most seeds, broomrape seeds are resistant to rapid microbial degradation due to phenols located in its testa (Cezard, 1973). However, hyphae of specific pathogens are able to penetrate the seed coat of broomrape dormant seeds, dissolving the endosperm cell walls and metabolizing the cytoplasm. The advantage of this approach using fungi is that it can be used in absence of host cultivation (Thomas et al., 1999). The parasitic weed radicle that emerges from germinated seed and carries the attachment organ is also targeted by those mycoherbicides (Abbasher and Sauerborn, 1992). This approach would inhibit parasitism by killing the young seedling before it attaches to the host root. The control of broomrape by mycoherbicides does not so far provide the level of control required in highly infested soils (Aly, 2007). Novel approaches can increase broomrape control by fungi. A “multiple-pathogen strategy” in which two or more pathogens are combined has been proved successful for the control of broomrape causing a synergistic effect that can lead to 100% broomrape control (Dor and Hershenhorn, 2003; Müller-Stöver et al., 2005). Refined formulations and encapsulations of fungal propagules increase efficacy in biocontrol by reducing desiccation or microbial competition (Amsellem et al., 1999; Quimby et al., 1999; Kroschel et al., 2000; Müller-Stöver, 2001; Aybeke et al., 2015). Engineering of virulence-enhanced mycoherbicides is another approach of great interest. This approach is based on the selection of naturally occurring mutants that overproduce and excrete an enhanced amount of specific amino acid with broomrape inhibition properties on seed germination and radicle growth (Vurro et al., 2006; Sands and Pilgeram, 2009).

##### Bacteria as biocontrol agents

*Pseudomonas aeruginosa, P. fluorescens, Bacillus atrophaeus, B. subtilis* are promising biocontrol agents targeting the growth of broomrape radicles (Barghouthi and Salman, 2010). In addition to this direct effect, ethylene-producing bacteria such as *Pseudomonas syringae* pv. *glycinea* induce ethylene-mediated suicidal germination in *Striga* sp. (Berner et al., 1999; Ahonsi et al., 2003), a close relative of broomrapes, however, broomrape germination is not responsive to ethylene (Joel, 2000). *Azospirillum brasilense* is reported to inhibit broomrape radicle growth (Dadon et al., 2004).

##### Microbial interactions that interfere on broomrape ability to recognize its host

Broomrape seeds are less capable to recognize crop roots colonized by arbuscular mycorrhizal fungi, *Rhizobium leguminosarum* or *Azospirillum brasilense* due to change in the composition of the root exudates in colonized plants (Dadon et al., 2004; Mabrouk et al., 2007a; Fernández-Aparicio et al., 2009c, 2010b; Louarn et al., 2012). A reduced content of broomrape germination-inducing factors in root exudates of mycorrhizal plants has been demonstrated (López-Ráez et al., 2011). Those interactions promote the broomrape seed bank remains dormant inhibiting the initiation of broomrape parasitism, and therefore its rates of seed bank replenishment.

#### Low-Inducers Crop Genotypes

Breeding for broomrape resistance stands out as the most economic, easy to adopt and environmentally friendly practice. Because parasitic weeds require host encoded molecules to stimulate the initiation of parasitism both at the level of seed germination and haustorium initiation, breeding for low-inducers genotypes of those processes are obvious targets for resistance (Yoder and Scholes, 2010). Sources of natural resistance based on low exudation of germination-inducing factors exist in legumes and sunflower and are highly effective in inhibiting broomrape weed parasitism (Labrousse et al., 2001, 2004; Rubiales et al., 2003b, 2009a; Pérez-de-Luque et al., 2005; Sillero et al., 2005; Abbes et al., 2010; Fernández-Aparicio et al., 2012b, 2014).

Sources of natural resistance based on reduced release of haustorium-inducing factors is a doubly interesting strategy to inhibit broomrape parasitism because not only it prevents broomrape parasitism in the current crop, but also it promotes the demise of the seed bank by promoting suicidal germination. Sources of low-inducers genotypes exist in crops species attacked by the close related parasitic weed *Striga* (Rich et al., 2004). It remains unknown whether host factors are required by broomrape radicle to initiate haustorium and consequently this strategy has not been fully explored.

### Control Strategies Targeting Host Penetration

The major strategy that specifically impedes the broomrape capacity to penetrate the host once the radicle has made contact with host root, is based on the use of host resistance, either genetic resistance obtained by breeding (Pérez-de-Luque et al., 2009; Yoder and Scholes, 2010), or induced resistance by abiotic or biotic agents (Gonsior et al., 2004; Kusumoto et al., 2007).

#### Resistant Crops to Broomrape Invasion

Depending on the genetic background of the resistant host, the intrusive cells of broomrape seedling can be stopped at three different levels in their way of penetration through the root layers to achieve connection with the host vascular system. The first barriers are imposed at the cortex level with reinforced cell walls mediated by either protein cross-linking or with the deposition of metabolites such as suberin, or callose. In addition, accumulation of toxic phenolic compounds at the infection point can be observed in some resistant varieties. Resistance that occurs in the endodermis is mediated by lignification of endodermal and pericycle cell walls. Resistance that occurs in the central cylinder is related with accumulation of phenolic compounds in the surrounding tissues and nearby xylem vessels inducing a toxic release near the parasite impeding vascular connection (Pérez-de-Luque et al., 2009). However, selecting for high phenolic varieties is likely to induce many other side changes altering agronomic performance.

Lack of knowledge in the molecular regulation of the host-parasite interaction during crop invasion has impeded the development of varieties carrying transgenes with capacity to inhibit broomrape penetration. As alternative, transgenic resistant crops have been engineered with broomrape-inducible expression of toxins specifically targeting the penetrating broomrape seedling. This strategy to abort broomrape invasion requires regulating the toxin production with promoters specifically induced around the site of *Orobanche* penetration such as the *HMG2* promoter, ensuring correct delivery of the toxic effect to the broomrape penetrating seedling and overall low concentration of the toxin in the rhizosphere. As a consequence the crop is protected from broomrape invasion (Joel and Portnoy, 1998; Westwood et al., 1998; Hamamouch et al., 2005; Aly et al., 2006).

#### Abiotic Inducers of Resistance

Induced disease resistance mediated by endogenous salicylic acid (SA) also described as systemic acquired resistance (SAR) induces hypersensitive responses in many plant species against fungal, bacterial and viral diseases. SA promotes resistance to broomrape. Abiotic inducers of SAR thus represent an innovative approach to control broomrape parasitism. Benzo-(1,2,3)-thiadiazole-7-carbothioic acid *S*-methyl ester (BTH) acts as a functional analog of SA and activates defense responses in susceptible hosts leading to lignification of the endodermis and a consequent inhibition to up to 98% broomrape parasitism (Gonsior et al., 2004; Pérez-de-Luque et al., 2004; Kusumoto et al., 2007). Commercially available as Bion^®^, field doses of 0.8 kg ha^–1^ are recommended to inhibit *P. ramosa* parasitism in hemp and tobacco (Gonsior et al., 2004), crops for which resistant varieties are not available. In other pathosystems, amino acids such as D-L-β-amino-*n*-butyric acid or L-methionine induce resistance in crop plants against pathogen attack. This resistance is coordinated with the expression of genes encoding for pathogenesis-related proteins (Sarosh et al., 2005; Hasabi et al., 2014). The ability of L-methionine to stop the entrance of broomrape intrusive cells into the host-root layers has not been studied. However, when Vurro et al. (2006) applied L-methionine in pots to tomato roots the number of broomrape seedlings that successfully developed parasitism was highly reduced. Though, the effect of L-methionine on internal crop resistance was not studied and requires further investigation. If this effect is confirmed, L-methionine use to elicit resistance to broomrape in susceptible crops could be a straightforward strategy either by direct applications of this amino acid in the soil as explained in Section “Control Strategies Targeting Host Penetration” or delivered by overproducing and excreting microorganisms as explained in Section “Strategies to Control Underground Broomrapes Acting after Establishment.”

#### Biotic Inducers of Resistance

Biotic inducers of systemic resistance have also proved being successful against broomrape parasitism under experimental conditions. *Rhizobium leguminosarum* induces defense mechanisms based on elevated induction of the phenylpropanoid pathway conferring mechanical and chemical barriers to the parasite penetration (Mabrouk et al., 2007a,b,c, 2010). Although the effect of jasmonic-acid-dependent induced systemic resistance (ISR) against parasitic plants is less clear (Kusumoto et al., 2007; Hiraoka et al., 2009; Yoder and Scholes, 2010), strains of *Pseudomonas* sp. inducers of ISR (Gozzo, 2003) and commercially available as Proradix^®^ can reduce broomrape parasitism by 80% in susceptible cultivars of hemp and tobacco without phytotoxic effect on the crop (Gonsior et al., 2004). Based on the results obtained in their greenhouse experiments, these authors recommended field doses of 1.6 kg ha^–1^ for crop densities of 32,000 tobacco plants ha^–1^. Unfortunately this technique represents another example of highly promising broomrape control strategy that has never been validated in field experiments.

### Strategies to Control Underground Broomrapes Acting after Establishment

Once broomrape has established connection with the vascular system of its hosts, broomrape management should be performed quickly to abort at earlier stages the strong parasitic sink for nutrients and water. Major feasible strategies for controlling broomrape and gain productivity in the current crop are those based on cultural practices that promote host scape to parasitic damage by improving host sink competitiveness, selective chemical control of the parasite via the haustorium, and host resistance based in physical, chemical barriers and physiological incompatibility. Some of the strategies discussed in previous sections such as biological control maintain their control action at post-attachment stages and will not be discussed again in this section.

#### Cultural Control of Post-attached Parasites

Delayed sowing date is a traditional method that can show high degree of success on inhibiting parasitism if implemented correctly (López-Granados and García-Torres, 1996; Rubiales et al., 2003a; Pérez-de-Luque et al., 2004; Grenz et al., 2005). This technique promotes the host plant to fulfill its required thermal time to flower in a shorter number of days, making the grain filling period shorter. The host reproductive sinks compete earlier and stronger against the parasitic sink and in consequence less nutritive resources are allocated to the parasite (Manschadi et al., 1996). However, the overall productivity of the host-parasite system is also reduced due to the shorter growing period being detrimental for crop yield. Therefore, decisions on the date of sowing has to be well-adjusted in order to balance the loss of productivity due to shorter growing period with gain of productivity due to reduced parasitism. Delaying sowing date has, however, a general drawback by reducing yield potential under normal development so that plant breeding program tend generally to favor long lasting cultivars with early sowing dates.

Besides date of sowing, nutrient management can promote both tolerance and increased resistance in crops to broomrape parasitism (Parker, 2009; Labrousse et al., 2010). Broomrape attack is more severe on crops growing in low fertility soils. Besides the effects of fertilization management on pre-attached broomrape stages described in previous sections, high soil fertility can induce crops to endure broomrape parasitism by helping the host to maintain a favorable osmotic potential that reduces the parasitic sink strength (Gworgwor and Weber, 1991).

#### Chemical Control of Post-attached Parasites

Four broomrape features define the post-attachment herbicidal strategy in comparison with non-parasitic weeds. First, broomrape weeds are achlorophyllous and therefore those herbicides that target photosynthetic process, e.g., triazines or substituted urease [C group in the Herbicide Resistance Action Committee (HRAC) classification], will have only limited effect on broomrapes. Second, broomrape weed exerts their damage underground right after attachment and therefore, contact herbicides applied after broomrape emergence, e.g., 2,4-D, had no effect on limiting yield loss in the current crop. Third, broomrape underground attachments do not take herbicides from the soil but only systemically from the host and therefore, this strategy is limited to systemic herbicides applied to herbicide-resistant crop varieties that do not metabolize the herbicide into inactive forms. And four, despite reports on broomrape inefficient machinery for nitrogen assimilation, and on amino acid fluxes from the host phloem to the parasite, herbicides inhibiting amino acid biosynthesis in the parasite via suppressive action on broomrape-encoded acetolactate synthase (ALS) and enol-pyruvylshikimate phosphate synthase (EPSPS) enzymes are able to kill broomrape.

Current chemical control of post-attached broomrape life stages is mainly achieved with foliar applications of systemic herbicides inhibiting ALS (imidazolinones, sulfonylureas) or EPSPS (glyphosate) to the leaves of crop varieties carrying target-site resistances to those herbicides to avoid direct injury of their metabolism. The target-site herbicide-resistance is based on a modification of the enzyme in such a way that it binds to its normal substrate in the amino acid biosynthesis pathway but not to the herbicide. This kind of resistance is more interesting than other mechanisms of resistance that usually involve translocation and enhanced metabolism, resulting in lower herbicide concentration in the sap of the host plant. With target-site resistance, the herbicide translocates unmetabolised to the underground broomrape via the haustorium inflicting its suppressive action in the parasite (Gressel, 2009).

Target-site resistances have been successfully developed in crops either by classical breeding such as sunflower, by screening mutagenized crop populations such as the case of oilseed rape or by transgenic techniques such as tomato, tobacco, carrots, and oilseed rape (Joel et al., 1995; Aviv et al., 2002; Slavov et al., 2005; Tan et al., 2005). Such target-site resistance is also available in other broomrape-susceptible crops but remains to be tested and registered to control broomrape. Engineered host crops harboring herbicide-resistance transgenes have not yet been commercialized for broomrape management (Gressel, 2009^2^).

An alternative to the selective use of herbicides when target-site resistance is not available for a specific crop is the touchy use of repeated applications of non-selective herbicidal doses to promote sublethal effects for the crop but lethal effects to the initial stages of post-attached parasitism (Foy et al., 1989). This strategy requires a careful calibration of doses and timing depending on the host crop and underground phenology of broomrape determined by local conditions and crop (Hershenhorn et al., 1998, 2009; Eizenberg et al., 2006). Systemic translocation of nanoencapsulated herbicides could improve this herbicidal approach (Pérez-de-Luque and Rubiales, 2009).

#### Resistant and Tolerant Cultivars

Death of the young broomrape tubercles shortly after nutritive flow initiation has been observed in cultivars carrying post-haustorial resistance in the form of growth arrest and necrosis of young tubercles. Several mechanisms underlying this resistance have been described at this stage such as production of gel-like substances within host vessels blocking the transfer of nutrients, host-encoded toxic-compounds delivered into the parasitic tissue though the vascular system and hormonal incompatibility that leads to abnormal haustorial maturation with scarce vascular connections (Fernández-Aparicio et al., 2008c; Pérez-de-Luque et al., 2008, 2009). Those mechanisms kill the broomrape either by inducing toxic effects or by starving the parasite.

Tolerant varieties are able to endure infection with minor losses on productivity. Crops that reach their seed filling period earlier than broomrape initiates its underground bud development are able to restrict parasitic sink and endure parasitic damage (Manschadi et al., 1996; Grenz et al., 2005; Fernández-Aparicio et al., 2009a, 2012a). In addition, some modifications of host biochemistry have been described in tolerant crops inducing low performance of the parasite when attached. High osmotic potential in roots and drop in amino acid levels in the phloem has been reported in tolerant varieties of faba bean in response to broomrape parasitism. The consequent reduced flux of water and nutrients toward the parasite, low utilization of host-derived sucrose and lower levels of soluble proteins limits the parasitic sink strength and yield losses due to broomrape parasitism (Abbes et al., 2009).

## Conclusion

If left uncontrolled during one or a few seasons, broomrape weeds build a hardly destructible seed bank in agricultural soils that further renovates at a rate of millions of seeds per ha each year a susceptible crop is infested. In addition, their mixed traits of weed and underground pathogen, make their control tricky. Even the easiest method of control, herbicides, requires broomrape specific-optimization for each cropping system to target the most vulnerable broomrape life stage, the young attachments while preserving the crop. It seems more and more obvious that a single strategy has low probability to control broomrapes. Instead an integrated control program including a battery of broomrape-specific measurements is preferable.

We have seen that several opportunities to stop the cycle of the parasite have been explored. The development of the solutions has usually not been conducted to their end so that many potential ways of controlling broomrape are not on the market. What we have often seen is that the solution has to propose a modification that makes the parasitic life cycle unfit to that of the crop. Still, as the parasite is synchronized on the crop development this means in some cases that the change disfavoring the parasite could also limit the maximum potential yield for the crop. As a consequence, except when deeply infested, the farmer (and thus the market) will not retain a solution that has economical negative drawbacks. This may well-explain why some several decades of parasitic plant research have not end up with satisfying and largely available tools for controlling this parasitic plant.

The advances yielded as intense research made connects the major critical steps of the life cycle of *Orobanche*, the external factors influencing it either through molecular dialog between the parasite and the crop or the soil and climatic environmental conditions naturally opens the way toward the potential effect of the cropping system in limiting broomrape parasitism: choice of the crop, timing, plant protection, soil perturbation, fertilization, etc. This lead us to build the list of the major feasible components that a model designed to quantify the effects of cropping systems on pest dynamics should include for specific broomrape control. Such a model would be a valuable tool to synthesize knowledge on broomrape life-cycle, to design and test management strategies and better predict the variability in effects observed for a given environment and set of agricultural practices. One future development would be to evaluate what could be the emerging risk at cultivating different crops, one of which may stimulate germination while the other offers opportunities for haustorium fixation. This would open the work on parasitism toward more community ecology and what can be considered the realistic nature of parasitism.

## Author Contributions

MF-A wrote the paper. XR and SG-L additional text, editing, and comments.

## Conflict of Interest Statement

The authors declare that the research was conducted in the absence of any commercial or financial relationships that could be construed as a potential conflict of interest.

## Footnotes

1 http://www.terresinovia.fr/orobanche/carte.php.

2 http://www.isb.vt.edu
